# Host Centrality in Food Web Networks Determines Parasite Diversity

**DOI:** 10.1371/journal.pone.0026798

**Published:** 2011-10-25

**Authors:** Tavis K. Anderson, Michael V. K. Sukhdeo

**Affiliations:** 1 Graduate Program in Ecology and Evolution, Rutgers University, New Brunswick, New Jersey, United States of America; 2 Department of Ecology, Evolution and Natural Resources, Rutgers University, New Brunswick, New Jersey, United States of America; University of California, Berkeley, United States of America

## Abstract

**Background:**

Parasites significantly alter topological metrics describing food web structure, yet few studies have explored the relationship between food web topology and parasite diversity.

**Methods/Principal Findings:**

This study uses quantitative metrics describing network structure to investigate the relationship between the topology of the host food web and parasite diversity. Food webs were constructed for four restored brackish marshes that vary in species diversity, time post restoration and levels of parasitism. Our results show that the topology of the food web in each brackish marsh is highly nested, with clusters of generalists forming a distinct modular structure. The most consistent predictors of parasite diversity within a host were: trophic generality, and eigenvector centrality. These metrics indicate that parasites preferentially colonise host species that are highly connected, and within modules of tightly interacting species in the food web network.

**Conclusions/Significance:**

These results suggest that highly connected free-living species within the food web may represent stable trophic relationships that allow for the persistence of complex parasite life cycles. Our data demonstrate that the structure of host food webs can have a significant effect on the establishment of parasites, and on the potential for evolution of complex parasite life cycles.

## Introduction

Food webs are abstractions of nature that describe community topology via networks of trophic interactions [1], [2]. The information provided by existing topological (who eats whom) webs has provided a fertile resource for the generation of theory on the determinants of community structure and the stability of ecosystems [1], [3], [4], [5], [6]. For example, the topology of a food web may help in understanding the flow of energy through systems and whether population dynamics are more or less stable in highly diverse communities relative to low diversity communities [6], [7], [8], [9]. Further, several topology-based metrics have become key parameters in the theoretical search for general patterns in food webs [7], and as determinants of food web stability [9].

Parasites have largely been understudied in these systems, and there have been few attempts to use the topology of the free-living host community to describe parasite dynamics [10], [11], [12]. However, highly resolved topological food webs [13], [14] show features of real structure that may be important in the persistence of complex parasite life cycles [15]. First, free-living hosts serve as both habitat and dispersal agents, and if transmission of a parasite is a function of the density of the final host, an abundance of hosts will result in an abundance of parasites [16], [17]. Second, because many parasites tend towards high host specificity [18], [19], increasing the diversity of host communities may result in a concomitant increase in the diversity of parasites [20], [21]. Third, trophically transmitted parasites are dependent upon the feeding habits of predators and prey for transmission [22]. Consequently, patterns of parasite diversity are contingent upon, and susceptible to, the structure and distribution of feeding interactions and the abundance of host in the free-living community [23]. In essence, the structure of the host food web is likely to exert a strong selective pressure on the evolution of parasite transmission strategies and subsequent patterns of parasite diversity observed in extant systems [23], [24], [25].

Clusters of species that have a critical place in the topology of the host network are likely to provide insight into the diversity of parasites in ecosystems for two reasons. First, those host species that fall into core clusters within ecological networks are likely to experience fewer fluctuations in abundance relative to those that fall in the periphery of a network [26] providing a reliable resource for parasites. Second, clusters of tightly interacting species that drive nestedness and modularity in food webs yield stable predator-prey trophic links [27], [28] and exploiting these stable links may ensure successful completion of the parasite life cycle. This is particularly important for helminth parasites with complex life cycles involving two or more hosts. As a general rule, transmission between the final intermediate host and the definitive host occurs via predation. The reliance on this form of transmission, over evolutionary time, is likely to have favored parasitism of host species that are central to the structure of food webs, and fall within interactions that are relatively “strong” (e.g. [12], [24]). Consequently, identifying patterns in the topology of ecological networks and linking regularities in the networks to parasite community dynamics is central to understanding how parasites establish and persist in host communities.

An ideal situation in which to test the effect of network topology on the emergent patterns of system parasite species richness would be within a series of islands of varying ages as in MacArthur & Wilson's classic island biogeography study [29]. MacArthur & Wilson posited that the number of species within a discrete and isolated system was a consequence of the species previously located there and the processes of immigration extinction and speciation. A consequence of this is that as a community assembles, the network and trophic structure of the food web should also change, revealing patterns of community complexity [30]. In this study, we use four brackish tidal marshes, each with a distinct host diversity and community composition as a surrogate for individual islands with a range of diversity and community complexity. Metazoan (helminth) parasites are common in invertebrates, fishes and birds in these marshes [31], and use a variety of vertebrate definitive hosts, molluscan first intermediate hosts, and fish second intermediate hosts. These life history strategies are intimately tied to the trophic interactions between free-living species, and as a result, the structure of the food web should act as a template for transmission. Using network centrality metrics [32] that describe the positional importance of free-living hosts, we quantify characteristics of the food web that are necessary for complex parasite life cycles to persist, and the potential mechanisms driving parasite species richness within host species. We report that the diversity of parasites within host species is largely determined by how well connected and central a host is within the network.

## Methods

### Ethics Statement

Field collections were conducted under scientific permits issued by the New Jersey Department of Environmental Protection, Division of Fish and Wildlife, Marine Fisheries Administration (#0558, #0628, and #0746) and Bureau of Freshwater Fisheries (#0536, #06-008, and #07-019). Prior to necropsy, fish were maintained briefly in aquaria following animal care protocols approved by The Animal Care and Facilities Committee at Rutgers University, Office of Research and Sponsored Programs (Protocol 00-012: NIH Assurance Number A3262-01). Fish euthanasia was conducted in accordance with the 2000 Report of the American Veterinary Medical Association Panel on Euthanasia, approved by The Animal Care and Facilities Committee at Rutgers University under the protocol described above (Protocol 00-012): fish were placed in a buffered 300 mg/L solution of tricaine methanesulfonate (MS-222) until cessation of opercula movement, followed by pithing of the brain and spinal cord.

### Defining the study sites

Sampling occurred within four brackish tidal marshes in the New Jersey Hackensack Meadowlands (USA): over 90% of the marshes in the Meadowlands are heavily impacted due to decades of anthropogenic disturbances [33]. These disturbances, largely in the form of tidal restriction and habitat fragmentation, have resulted in marsh habitats dominated by *Phragmites australis* (common reed); a plant whose presence is typically an indicator of habitat degradation [34]. Recent large-scale restoration projects with the goal of creating and enhancing a variety of marsh habitats for wildlife, and to bring about the recovery of wetland function [35], have created spatially delineated habitats that vary in time since restoration: Oritani marsh (unrestored); Mill Creek marsh (20 years since restoration); Harrier Meadow (10 years); Secaucus High School marsh (0 years).

Mill Creek marsh (20 year) is a 57-hectare tidal marsh bordered by highways and residential land (40°47′45″ N 74°02′30″ W). The marsh restoration has resulted in low marsh habitats dominated by *Spartina* sp and *Distichlis* sp that are flushed daily by the tides: tidal impoundments and lowland scrub-shrub habitats lay along the marsh/upland ecotone. Harrier Meadow marsh (10 year) is a 32-hectare tidal marsh surrounded by tidal mudflats and urban development (40°47′12″ N 74°07′3″ W). The marsh has low marsh habitat similar in vegetation to Mill Creek, shallow open water impoundments that are hydrologically connected to the surrounding mudflats, areas of higher elevation dominated by *Phragmites australis, Lythrum salicaria,* and lowland scrub-shrub habitats. Secaucus High School marsh (0 year) is a 43-hectare tidal marsh bordered by a river and residential development (40°48′17″ N 74°02′52″ W). The site is currently dominated by the common reed (*P. australis*), and contains narrow sinuous channels, several mosquito ditches, and tide gates. Tidal flow is restricted and large sections of the marsh receive rare inundation at high tide: restoration to restore regular tidal flow, and wetland function are currently underway. Oritani marsh (unrestored) is a 224-hectare tidal marsh that has no record of human alteration or use (40°47′57″ N 74°05′07″ W). The marsh is undeveloped and includes more than 150 hectares of upland area and a smaller area of high and low marsh with small tidal channels. The upland areas are dominated by a dense monoculture of common reed (*P. australis*). The high marsh areas are dominated by saltmarsh hay (*Spartina patens*), while the low marsh areas are predominately smooth cordgrass (*S. alterniflora*), marsh fleabane (*Pluchea pupurascens*), and dwarf spikerush (*Eleocharis pavula*).

Although the ‘real’ food web is likely to span the entire New York-New Jersey estuary complex, we constructed four food webs that are constrained by physical boundaries (roads, urban development) that surround each marsh site. In addition, we limit the food webs to those species found in tidally influenced sediment and the vegetated habitat within the marsh (*sensu* [36]). By constraining each food web spatially, we omit birds, mammals, and invertebrates that are transient in the marsh habitat. Further, we do not consider the edge of each marsh, and the species located within this habitat, as part of our community as these species are likely indicative of the mudflats in the estuary complex, or the urban development that surrounds each site.

Our preferred taxonomic unit for constructing each food web was species, although we were limited by our source data. As a consequence, some members of our food web were lumped into large categories (e.g. Nematoda, copepods, ostracods). Where possible, we empirically validated literature records for birds (point count surveys every three months starting in December 2005 and ending in December 2007: bird species were recorded if visually detected within a five-minute period at any of three survey stations within each marsh), benthos (benthic cores were taken at a depth of 5 cm every three months starting in December 2005 and ending in December 2006 at three locations within each marsh), and fishes (active seine netting, minnow trapping, and trap nets were deployed every three months starting in December 2005 and ending in December 2007). However, the majority of species we document in our food webs were based upon community data collected from the literature for birds [37], [38], fishes [39], and benthos [40], [41]. We included species from these records if they comprised more than 0.5% of the individuals sampled, but relaxed this criteria for top predators given their relative importance in the structure of food webs and role as potential definitive hosts for parasites (*sensu* [36]). For basal species we lumped: terrestrial and aquatic detritus; micro and macroalgae; and the producer component of the food chain together. Though this represents a gross simplification of a high level of diversity [42], it has been used in other parasite food web studies as a method of minimizing complexity that may not be relevant in parasite transmission [36]. These criteria were used consistently for each food web: the species that fulfil these rules are listed in tables provided in the supplementary files (Table S1: Appendix S1).

### Food web topology

Food webs consist of a predator(i)-prey(j) matrix with *n* species, and were constructed following the methods in Cohen *et al.* [2], [43]. Given our interest in the topology of the free-living host network, we did not construct parasite subwebs *sensu* Lafferty *et al.* [36]. Consequently, our matrices and analyses were limited to traditional predator-prey interactions. Binary entries in these matrices indicate whether a predator eats a prey species. Trophic links were determined for all taxa using primary publications and monographs [44], [45], [46]. In cases where the diet description was overly vague (e.g. benthic invertebrates) we used our discretion, based upon adult body-size relationships, in assigning trophic links [47], [48]. We further extended links between predators and prey by inferring links using our empirical parasite records. Given that parasites are a useful indicator of host diet [49]: the presence of a parasite species within a host provides a robust indicator of host diet [22], [50]. Thus, a host species that serves as an intermediate host for a parasite species found in a specific predator will be a prey item for that predator [22].

Food web metrics were calculated for each predator-prey matrix and included the number of species (*S*), the number of observed links (*L_o_*), the number of potential links (calculated as the number of cells in the matrix, *S^2^*), linkage density (*d*), directed connectance (*C*) [51], and nestedness (N) [52]. Connectance (*C*  =  *L_o_/S^2^*) is the number of realized links (*L_o_*) divided by the number of possible links (*S^2^*). Measured in this way, *C* is the average fraction of species in a community consumed by the average species. Nestedness describes the extent to which a group of specialist consumers feed upon a subset of the prey eaten by generalists. To estimate nestedness we calculated matrix temperature using the software ANINHADO [53] that compares the extent to which a matrix is significantly nested relative to a series of null model generated matrices. The null model used to assess significance was implemented as Ce in ANINHADO. To allow for across network comparisons we also calculated relative nestedness [54].

Topology is a concept from graph theory that is used to characterise the structure and status of a network. To this end, we calculated features such as node degree, eigenvector centrality, betweenness, closeness and modularity. The degree (or connectivity; *k*) of a node, describes the number of links a singular node makes with other nodes and provides a fundamental metric. Using these values we calculated the cumulative degree distribution, a representation of the fraction of trophic species *P(k)* that have *k* or more trophic links. We examined these distributions by fitting three different models and ranked model fit using the Akaike Information Criterion [55]: (a) exponential *P(k)* ∼ exp(-γ*k*); (b) power-law *P(k)* ∼ *k* ^-γ^; and (c) truncated power-law *P(k) ∼ k^-^* ^γ^ exp(- *k/k_x_*). Eigenvector centrality scores correspond to the values of the first eigenvector of the predator-prey matrix, and may be interpreted as arising from a reciprocal process in which the value for each species is proportional to the sum of the centralities of those species to whom it is connected [56]. This implies that species with high eigenvector centrality values will be those that exist in densely populated substructures in the food web. A corollary of eigenvector centrality is the value of betweenness, a quantitative measure for describing the centrality of species, provided as the frequency with which a node is located on the shortest path between all other species [56]. Conceptually, those species with high-betweenness are those that represent “bridges” within the food web. Closeness provides a measure that describes the relative distance from a focal species to all other species. Intuitively, closeness provides an index of the extent to which a given species has short paths to all other species. These tests were computed in R v2.12.1 statistical programming language [57] with the sna: tools for network analysis package v.2.0.1. [58]. We measured an additional descriptive metric of network centrality using models of core/periphery structure [59]. The idea of network core/periphery structure in food webs is that there is a physical centre of the food web (species with high levels of interspecific interactions) and a periphery of a cloud of points in Euclidean space (species with fewer direct and/or indirect interactions). To estimate the core/periphery structure within each network we used UCINET 6. Last, we measured the modular structure of each food web using a clustering algorithm to define group-membership [60]. The algorithm, proposed by Allesina & Pascual [60], merges two important concepts: first, it identifies compartments (sets of highly interacting species), and secondly forms groups using these data and metrics that describe the similarity of species “roles” (sets of species that have similar interaction patterns).

### Field collections and incorporating parasites into the networks

Information on helminth parasites came from field sampling of a focal species, *Fundulus heteroclitus,* and a literature review of potential parasites of the free-living organisms present in the study system. *Fundulus heteroclitus* was selected as a focal species because it is a highly abundant resident marsh species along the east coast of North America, likely plays an important role in marsh food webs, and has a wide range of possible helminth parasites [61]. The abundance of *F. heteroclitus,* and its helminth parasites were measured every three months starting in December 2005 and ending in December 2007 (eight contiguous seasons: two fall, two spring, two winter, two summer). Fish were collected using a 4 mm seine and baited minnow traps; all habitats within each marsh were sampled for at least 5 days each season. From each seasonal collection, thirty fish were identified to species, euthanized and immediately necropsied. Fish necropsy was done using standard parasitological techniques. Helminth parasites collected during necropsy were identified using keys and primary literature. In addition to these empirical data, we selected twenty one representative parasites, that ranged in life cycle strategy and host specificity, and were likely to be found in each marsh site given the presence of particular hosts (see Table S2). Consequently, host-parasite links in these analyses were only included in the web when the parasite was known to have suitable hosts present for each life stage of the parasite species. Thus our network is not a comprehensive host-parasite network, but a subset of parasites within a network of host interactions.

### The topological determinants of parasite diversity

To test whether network topology affects the diversity of parasites within a host, we used regression tree analysis (RT). Regression tree analysis develops a set of ‘rules’ derived from predictor variables that best recreate the observed pattern in the response variable [62], [63]. The response variable in this analysis is parasite diversity within a host; predictor variables were topological food web metrics (Table 1). In this technique, trees are constructed by repeatedly splitting variables along binary nodes using predictive covariates that lead to an average value of the response variable. Nodes of covariates may be nested, with the most basal explaining the largest proportion of variation in the response variable. A major advantage of this analysis technique is that it does not rely on the assumptions that are required for the appropriate use of parametric statistics (i.e. Gaussian distribution of predictor variables), nor does it make assumptions about spatial or temporal autocorrelations. Further, regression tree analysis is not restricted by linearity in predictor and response variables or by multicollinearity in predictor variables. To avoid over-parameterization, trees were selected using the cost-complexity algorithm, whereby auxiliary nodes are cut if no significant loss in the mean square error of the predictions is detected. These trees were constructed with R v2.12.1 statistical programming language using the rpart: recursive partitioning package [64]: variable importance was determined using the caret: classification and regression training package [65].

**Table 1:** Predictors used in regression tree and random forest model building.

*Code*	*Description*	*Range*
Eigenvector	The value for each species is proportional to the sum of the centralities of those species to whom it is connected.	0.0151–1.00
Betweenness	The frequency with which a node is located on the shortest path between all other species.	0–79.26
Closeness	The relative distance from a focal species to all other species.	0.38–0.74
Degree	The number of links a singular species makes with other species.	1–73.00
Group	Group membership	1–19
Coreness	The relative distance from a focal species to the centre of the food web.	0.002–0.36
Marsh diversity	Species richness	71–122
Trophic generality	Trophic generality (*G*)	0–55
Trophic vulnerability	Trophic vulnerability (*V*)	0–70

To validate the structure of the generated regression tree, we use random forest methods to generate class predictions based on several regression trees. In brief, a series of regression trees are constructed using a random selection of some of the input predictor variables. A final tree is built, where the predictions are based upon the aggregate outcome of all the randomized trees forming the random forest [66]. In these analyses, we use fully cross-validated regression trees, and random forests with 1000 trees were used to predict parasite diversity within hosts. We analyzed all food webs together and separately and determined variable importance using R v.2.12.1 and the randomForest package [67].

Our *a priori* hypothesis was that the diversity of complex life cycle parasites would be higher in those host species that are highly connected, and fall within densely populated substructures of the food web i.e. eigenvector centrality score will be the most basal node in the regression tree.

## Results

### Structure of the free-living web

**Figure 1:**
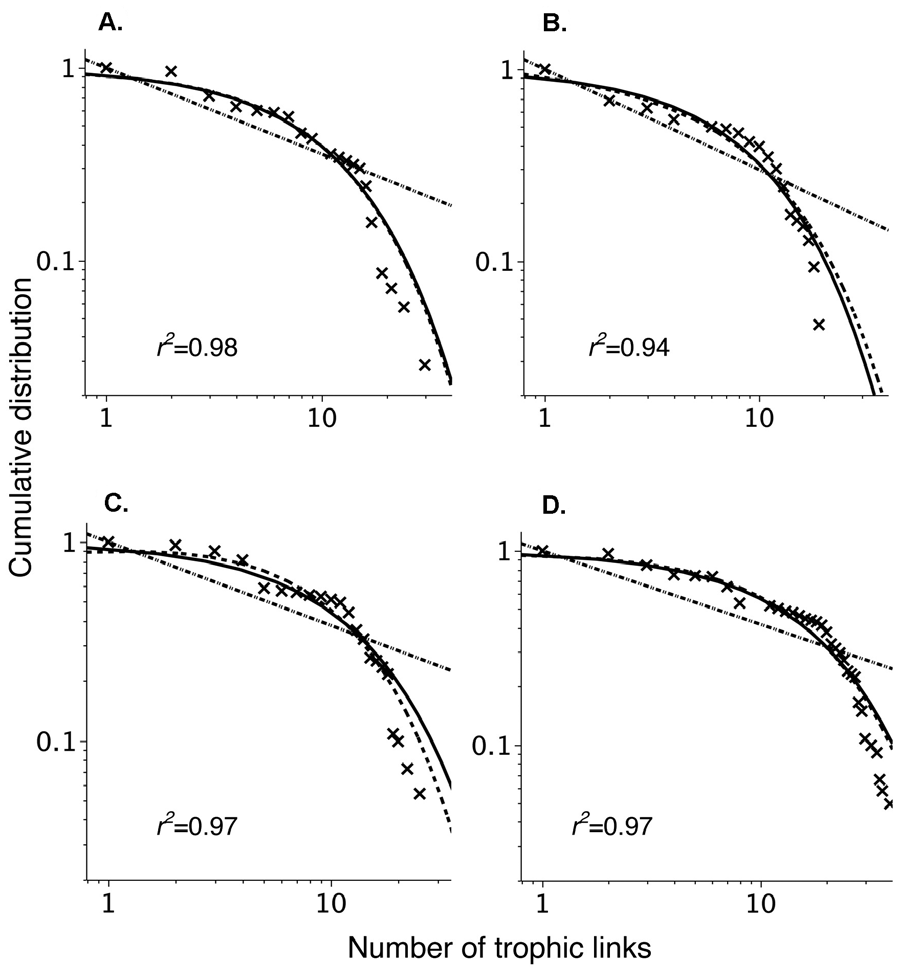
Log-log plots of cumulative distribution of links per species. (a) Oritani Marsh (unrestored), (b) Secaucus Marsh (0 year), (c) Harrier Marsh (10 year), and (d) Mill Creek Marsh. Cross marks represent observational data lines, and *r^2^* values represent the fit to the data of the best simple models: power-law distribution (straight line), truncated power-law distribution (downward curved dashed line), or exponential distribution (downward curved solid line).

The Oritani marsh (unrestored) included 71 species, and had 5041 potential links of which 629 were realised, resulting in a connectance of 0.125 (Table 2). The Secaucus Marsh (0 year) included 87 species, and had 7569 potential links of which 627 were realised, resulting in a connectance of 0.083 (Table 2). The restored marshes Harrier Marsh (10 year) and Mill Creek (20 year) included 112 and 122 species respectively; the resulting values of connectance were 0.096 for Harrier Marsh and 0.124 for Mill Creek Marsh (Table 2). All four of our trophic food webs displayed cumulative degree distributions that were different from what would be expected if the link distribution were random (Figure 1). Each food web had data that were consistent with an exponential (*AICc*  =  –115.54) or truncated power-law distribution (*AICc*  =  –113.52): as measured by *AICc* there was no difference in fit between these models (Δ*AIC*  =  2.02), though the data was not well represented by the power-law (*AICc*  =  –49.25). Good fits of the data to a power-law distribution were achieved in the range of 1-10 interactions per species (Figure 1), this was followed by a sharp cut-off for species with more than 10 interactions, resulting in a poor model fit [68], [69]. The identity of the best-fit model is secondary to our data departing from a power-law distribution; this suggests that super-generalist species are more rare than would be expected if the networks were built using a scale-free distribution to describe the number of interactions per species. Like many aquatic ecosystems, the food web had high diversity in the low and high trophic levels and with relatively few species in the intermediate trophic levels. The linkage density increased though not markedly so across the gradient of time post-restoration (Table 2).

**Table 2:** Summary of food web metrics for each of the estuarine food webs.

Parameters:	*Oritani Marsh (unrestored)*	*Secaucus Marsh (0 year)*	*Harrier Marsh (10 year)*	*Mill Creek Marsh (20 year)*
Number of species; *S*	71	87	112	122
Potential no of links; *S^2^*	5041	7569	12544	14884
Observed no of links; *L_o_*	629	627	1206	1846
Linkage density; *d*	8.86	7.21	10.77	15.13
Connectance; *C*	0.125	0.083	0.096	0.124
Relative nestedness; n*	0.75	0.75	0.86	0.81
Number of groups; *k*	15	15	18	19
Minimum *AIC_Groups_*	1361.204	1403.699	1851.464	3016.614

Statistics include species richness (*S*), potential links (*S^2^*), observed links (*L_o_*), linkage density (*d*), connectance (*C*), relative nestedness (n*), and number of groups yielding the minimum *AIC* for the group-based model described in the main text (*k*).

**Figure 2:**
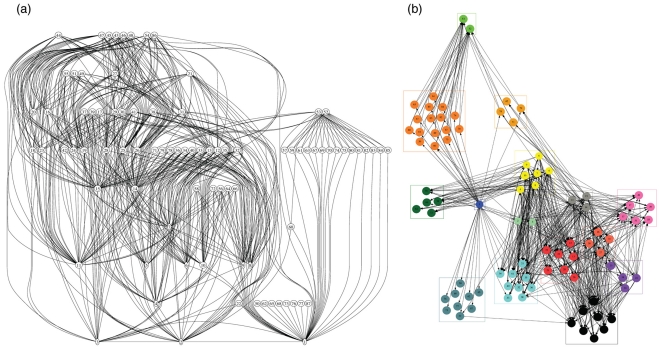
The structure of the food web at Secaucus High School Marsh. The marsh food web (a) without grouping, and (b) with species sorted according to their group affinity. The lines connect a consumer with a consumed species; the nodes represent species identified in Table S1. The grouping configuration is determined by [60], assessed using *AIC*, a configuration containing 15 groups was the best fit of the data. The grouping algorithm, seeks to partition the species into groups that make the density of connections within each sub-matrix maximal/minimal. Alternate group configurations are presented in Table S3.

All networks were significantly nested in comparison to randomised matrices (p<0.001; Table 2). We report the minimum *AIC* found by using the simple group based model [60] that determined that a configuration that contained 15 groups for Oritani and Secaucus Marshes, and 18 and 19 for Harrier Meadow and Mill Creek Marshes fit the data best (Table 2: Figure 2). Alternate group size configurations and their respective *AIC* values are contained in supplementary tables (Table S3).

### Parasite community in Fundulus heteroclitus

A total of 960 sentinel fish were studied: 30 collected in each of the eight seasons between 2006-07 in each of the 4 marshes. Ten taxa of metazoan parasites were identified including nematodes *Dichelyne bullocki* and *Contracaecum* sp; the digenean *Lasiotocus minutus* and metacercaria of *Ascocotyle diminuta Posthodiplostomum minimum*; monogeneans *Fundulotrema prolongis* and *Swingleus ancistrus*; acanthocephalans *Paratenuisentis ambiguous* and *Southwellina hispida* (cystacanth); the copepod *Ergasilus funduli*; these taxa infected more than 70% of the mummichogs examined. Parasite intensity per host ranged from 1 to 127.

### Where are the parasite life stages?

Our *a priori* prediction was that the diversity of complex life cycle parasites would be higher in those host species that are highly connected, and fall within densely populated substructures of the food web i.e. the regression tree analysis would support eigenvector centrality as the predominant factor in determining parasite diversity within a host. Using empirical parasite data from our field collections of *Fundulus heteroclitus,* and sampling of twenty-one parasite species from primary literature, we find that the best predictors of parasite diversity within a host are trophic generality, eigenvector centrality, and closeness (i.e. they are considered important variables in both regression tree and random forest models: Table 3). In regression trees, the calculation of variable importance is the reduction in the loss function (e.g. mean squared error) attributed to each variable at each split summed across the full tree. Consequently, a variable that does not appear as a node in the tree may explain more of the variability in the response variable than a predictor identified as a node. For the full regression tree models (Table 3: Table S4: Figure S1), the variables that are considered most important are closeness, coreness, eigenvector centrality, and trophic generality. For the random forest tree models (Table 3: Table S4: Figure S2), the variables that are considered the most important are trophic generality, eigenvector centrality, closeness, and trophic vulnerability.

**Table 3:** Regression tree and random forest model results.

*Model type*	*Site*	*R^2^*	*Most important variables in model*
Regression tree	All	52.97	Closeness, coreness, eigenvector, trophic generality
	Mill Creek	47.71	Closeness, trophic vulnerability, eigenvector, group
	Harrier Meadow	52.06	Trophic generality, eigenvector, closeness, betweenness
	Secaucus High School	46.88	Trophic generality, degree, coreness, eigenvector
	Oritani	33.86	Trophic generality, trophic vulnerability, degree, closeness
Random forest	All	47.39	Trophic generality, eigenvector, closeness, trophic vulnerability
	Mill Creek	21.88	Trophic generality, eigenvector, group, trophic vulnerability
	Harrier Meadow	42.71	Trophic generality, eigenvector, closeness, trophic vulnerability
	Secaucus High School	55.77	Trophic generality, closeness, eigenvector, group
	Oritani	3.94	Trophic generality, eigenvector, group, betweenness

The r^2^ value indicates the ability of the model to predict parasite diversity within an host. Also included are the four most important variables from the models listed in order of importance. See supplementary files for a regression tree graphic.

## Discussion

These data suggest that food web structure plays a significant role in the persistence of complex parasite life cycles and the diversity of parasites within free-living species. The key insight provided by our analyses is that the highly connected species within a food web, along with modular network structure, are likely to provide clusters of interactions that allow for higher transmission efficiency in trophically transmitted parasites. Clusters of interactions are particularly important for parasites with complex life cycles as they rely on feeding interactions between trophic levels, a strategy with a failure rate that is potentially offset by strong trophic links and transmission within food web compartments. Recent studies of parasitism in food web networks have also documented the increased use by parasites of free-living species that occupy central locations in the food web, and in free-living species that tend to have more predators [12], [70]. These data suggest that over evolutionary time, parasite species might become embedded in subsets of hosts, or clusters of hosts that ensure high transmission within the food web.

Several members of the free-living community, characterized by broad diets and high centrality scores, have significantly higher diversities of helminth parasites. Functionally, species that fall close to the centre of a food web (i.e. high closeness and eigenvector centrality scores), are best placed to accumulate resources and energy from lower trophic levels [71]. Further, those species with a broad diet are likely to ingest species that act as intermediate hosts for a diverse range of parasites, and consequently harbour higher within-host diversity [70]. One reason why there may be a reliance on such hosts is because species that fall at the periphery of the food web, or outside of tight clusters of interactions, are more susceptible to extinction [26], [72]. Consequently, parasite species that rely on hosts that are central to the food web are less likely to be subject to fluctuations in host availability and as such, increase the probability of successful transmission.

A second consideration is that the majority of trophically transmitted parasites fall within densely populated substructures in the food web. These link-dense areas (i.e. species with high eigenvector centrality scores) represent clusters of species that are linked more tightly together than they are to species in other areas of the network. These areas, and the interactions they document, form the basis for the “groups” or “compartments” we describe (Table 2: Table S3). Discussions of compartmentalization in ecological networks began in the 1960s, and despite some concerns [73], the presence of distinct compartments in food webs has been directly correlated with measures of system robustness [74], [75], [76]. Furthermore, highly resolved data sets reveal that many networks are highly cohesive, with several small groups of species connecting to a single dense core which plays a central role in determining network structure [74], [75]. A significant consequence of network cohesiveness is that the network may become more robust to perturbation, as changes are restricted to one area of the network. Notably, it has been demonstrated in population-level models that if a pathogen enters a particular compartment, the spread of that pathogen may be enhanced within these clusters of tightly interacting species [77]. Though not entirely analogous, it is likely that compartmentalization in food webs also facilitates the transmission of complex life cycle parasite species because transmission within a cluster of species is easier than transmission between clusters of species.

A fundamental aspect of searching for clusters of interactions is describing the distribution of feeding links in food webs. The appearance of a characteristic single-scale distribution of feeding links in our networks may be related to how these brackish marsh communities have assembled. It is likely that the mechanisms that produce the link distribution in our food webs differs from those that produce scale-free distributions observed in real world networks [68]. This is largely due to the violation of two assumptions in amenable models of real world networks: (i) the network grows at each time step through the addition of nodes and links and (ii) there is a preferential attachment of new nodes to other nodes with a higher number of links [78]. Predator-prey webs appear to violate the first assumption through the processes of immigration, extinction, and speciation [79]. Secondly, although there is yet to be a general consensus as to how new species link to existing species in food webs, it appears that immigrants do not always link to the most linked species [30], [80]. In an explicit test of the preferential attachment model, Olesen *et al.* [80] determined that the assembly process in a plant-pollinator network was intermediate between preferential attachment and random; with attachment constrained by the ecology (i.e. abundance, phenophase length) of the system. This is supported by our data, and a larger analysis of 16 food webs [8] that suggest there are fewer super-generalists than would be expected if new species preferentially attached to other highly linked species. The proposition that there are a few super-generalists that are driving the structure of the entire web is supported by the high degree of nestedness for each of our marsh food webs. These data imply that there is a distinct group of generalist species that interact amongst themselves and that there is a tendency for specialist species to interact with the most generalist species. This topological property has become a standard measure in food web analyses because of the potential for core generalist species to drive the evolution of entire systems.

Complementing the distribution of feeding links, and the generalist-specialist dichotomy we observed in our networks, is the presence of distinct groups of highly interacting species which we identified using the algorithm proposed by Allesina and Pascual [60]. The presence of such groups may have a significant effect on the coevolutionary process, and has been discussed in plant-pollinator systems [81], and as a potential stabilizing force in food webs [76]. In the case of our estuarine food webs, the observed groups represent tight clusters of feeding interactions that act as transmission routes for trophically transmitted parasites. The interaction between parasite and host is intimate and persistent, and there has been considerable selection for parasite stages to exploit host species that increase the probability for life cycle success. In some cases, parasites have circumvented diffuse predator-prey interactions by modifying the behaviour of intermediate hosts to make them more susceptible to predation from specific definitive hosts [82]. Though this is a fruitful approach to increasing transmission efficiency, it is not a predominant mechanism (reviews in [83]), and it is more likely that it is the structure of the host food web that exerts a stronger selective force on parasite life cycles [18], [19]. Consequently, it is likely that through evolutionary time, parasite species become embedded in groups of hosts that ensure high transmission. Those highly connected species in our food webs are heavily parasitized because they potentially provide a stable coevolutionary unit that complex life cycle parasites may exploit during their evolution and persistence [71].

The demonstration of modularity in these four estuarine food webs has implications for ecology and evolution outside of parasite transmission and life strategies. To our knowledge, there are few studies that have found modularity in food webs [76], [81], [84], though this is likely the result of poorly resolved data and the lack of sufficiently strong algorithms to detect modules. As the resolution of food web data improves (see [11]) and studies begin to incorporate module-detecting algorithms from the social sciences [59] and physics [85] it is likely that network modularity will be revealed as a critical component in the functioning of ecological networks, particularly with regards to the stability of ecological systems [5]. Indeed, recent work has demonstrated that compartmentalization may significantly increase the likelihood of food web persistence [86]. Further, the identification of modules of species within networks may reveal critical information about the effect of species extinctions on community dynamics, the impact of exotic species on native plants and animals, the spread of infectious diseases within and between communities, and potentially provide the critical units of tightly interacting species that could operate as coevolutionary units [87].

One assumption of our study is that our selective sampling of parasites, and the patterns that emerge, are representative and can be extended to parasites in general. Though our interpretation is intuitive, and supported in part by similar findings in other estuarine food webs [12], [70], it is potentially a result of sampling only 25 parasite species. Our analyses may be biased for two reasons: firstly, information on parasites is typically more detailed for common and charismatic host species; secondly, systematic parasitological sampling of our study region is incomplete, and as such, we have included parasites based upon host records from distant locations (i.e. California and Europe). That said, our study falls within the bounds of previous studies, such as Thompson *et al.* [88] who explored the role of nine parasite species in a food web network, to Lafferty *et al*. [36] who developed *de novo* a host-parasite food web that included 33 helminth parasites. Though our approach may have resulted in an overestimation of parasite diversity in certain species, the approach we have taken to including parasite species and the subsequent extrapolation to generate hypothesis for further testing is appropriate.

To conclude, the analytical food web framework was formally introduced in the early Twentieth century and has since developed into a widely appealing and accepted approach to describing species interactions. While debate continues about the utility of food webs as synthetic tools it is plausible to suggest that at the very least, highly resolved food webs provide an opportunity to integrate processes operating at the level of the free-living community with those important for parasites. Indeed, previous studies have documented how parasites permeate entire ecosystems; positions derived from the frequency of complex life cycles, with one parasite species interacting with many free-living hosts substantially altering food web metrics [11]. More importantly, our study has demonstrated how food web structure strongly influences parasite diversity patterns, a result of the dependence of parasites upon their free-living hosts and the nature of the ecological network in which they reside.

## Supporting Information

Figure S1 Pruned regression tree analysis of within-host parasite diversity. The explanatory variables were trophic generality (num_prey), trophic vulnerability (num_pred), eigenvector centrality, closeness, group membership, marsh diversity and coreness. Each node is labelled with the mean parasite diversity, and number of observations in the group. Further, each of the splits (nonterminal nodes) is labelled with the variable and its values that determine the split. The tree explained 52.97% of the total sum of squares, and the vertical depth of each split is proportional to the variation explained.
(DOC)

Figure S2 Random forest variable importance. **(a)** Determined by calculating the mean square error during each random permutation (n = 1000), and determining the difference between the average value and the prediction error on the out-of-bag data; and **(b)** the total decrease in node impurities from splitting on the variable averaged across all trees (n = 1000).
(DOC)

Table S1 List of taxa and species codes in the Mill Creek, Harrier Meadow, Oritani, and Secaucus High School Marsh food webs.
(DOC)

Table S2 Life cycle characteristics of select parasites in the Meadowlands estuary complex. Parasite species marked with a star (*) represent those identified in field collections of *Fundulus heteroclitus.*
(DOC)

Table S3 Arrangement of Mill Creek, Harrier Meadow, Oritani and Secaucus High School Marsh food webs into group structure by the algorithm proposed by Allesina & Pascual [60].
(DOC)

Table S4 Regression tree variable importance determined by the summed reduction in the loss function (e.g. mean squared error) attributed to each variable at each split. Random forest variable importance is determined by calculating the mean square error during each random permutation (n = 1000), and determining the difference between the average value and the prediction error on the out-of-bag data.
(DOC)

Appendix S1 Food web adjacency matrices for Mill Creek, Harrier Meadow, Oritani, and Secaucus High School Marsh.
(XLS)
