# Supplementary material for: Host Centrality in Food Web Networks Determines Parasite Diversity
Source: PLoS One. 2011 Oct 25;6(10):e26798. doi: 10.1371/journal.pone.0026798 (PMC3201966; doi:10.1371/journal.pone.0026798)
Supplement: Figure S1 — Pruned regression tree analysis of within-host parasite diversity. The explanatory variables were trophic generality (num_prey), trophic vulnerability (num_pred), eigenvector centrality, closeness, group membership, marsh diversity and coreness. Each node is labelled with the mean parasite diversity, and number of observations in the group. Further, each of the splits (nonterminal nodes) is labelled with the variable and its values that determine the split. The tree explained 52.97% of the total sum of squares, and the vertical depth of each split is proportional to the variation explained. (DOC) [file pone.0026798.s001.doc]

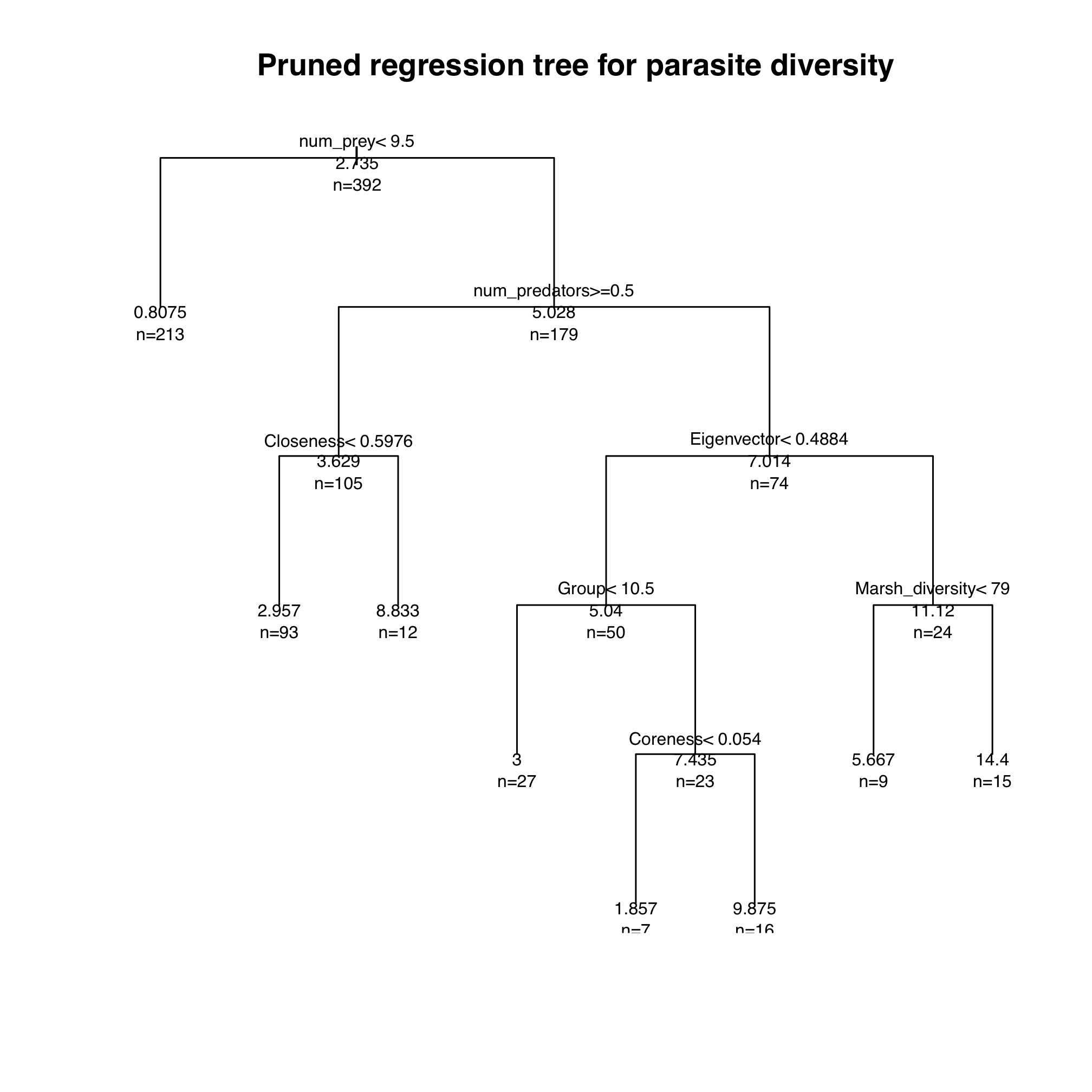


Figure S1. Pruned regression tree analysis of within-host parasite diversity. The explanatory variables were trophic generality (num_prey), trophic vulnerability (num_pred), eigenvector centrality, closeness, group membership, marsh diversity and coreness. Each node is labelled with the mean parasite diversity, and number of observations in the group. Further, each of the splits (nonterminal nodes) is labelled with the variable and its values that determine the split. The tree explained 52.97% of the total sum of squares, and the vertical depth of each split is proportional to the variation explained.
