# Supplementary material for: Host Centrality in Food Web Networks Determines Parasite Diversity
Source: PLoS One. 2011 Oct 25;6(10):e26798. doi: 10.1371/journal.pone.0026798 (PMC3201966; doi:10.1371/journal.pone.0026798)
Supplement: Figure S2 — Random forest variable importance. (a) Determined by calculating the mean square error during each random permutation (n = 1000), and determining the difference between the average value and the prediction error on the out-of-bag data; and (b) the total decrease in node impurities from splitting on the variable averaged across all trees (n = 1000). (DOC) [file pone.0026798.s002.doc]

**A.**

**B.**


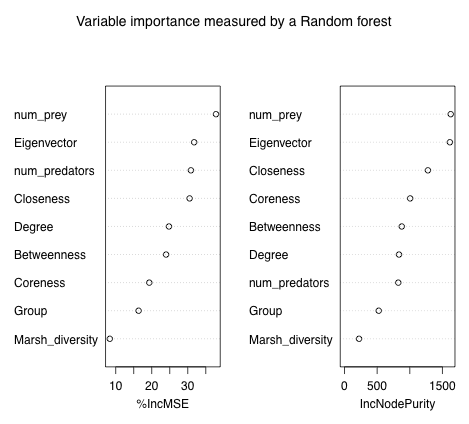


Figure S2: **(a)** Random forest variable importance, determined by calculating the mean square error during each random permutation (n = 1000), and determining the difference between the average value and the prediction error on the out-of-bag data. **(b)** The total decrease in node impurities from splitting on the variable averaged across all trees (n = 1000).
