# Supplementary material for: Host Centrality in Food Web Networks Determines Parasite Diversity
Source: PLoS One. 2011 Oct 25;6(10):e26798. doi: 10.1371/journal.pone.0026798 (PMC3201966; doi:10.1371/journal.pone.0026798)
Supplement: Table S1 — List of taxa and species codes in the Mill Creek, Harrier Meadow, Oritani, and Secaucus High School Marsh food webs. (DOC) [file pone.0026798.s003.doc]

Table S1: List of taxa in the Mill Creek Marsh, Harrier Meadow, Oritani, and Secaucus High School food webs.

| **Site** | **Taxon** | **Common name** | **Code** |
| --- | --- | --- | --- |
| Mill Creek Marsh | *Benthic invertebrates* |  |  |
|  | *Anisolaris maritime* |  | 1 |
|  | *Amphipoda* spp. |  | 2 |
|  | *Annura maritime* |  | 3 |
|  | Aranae sp. 1 |  | 4 |
|  | *Balanus improvisus* |  | 5 |
|  | Bryozoan sp. 1 |  | 6 |
|  | *Callinectes sapidus* |  | 7 |
|  | Chironomidae sp. 1 |  | 8 |
|  | *Congeria leucopheata* |  | 9 |
|  | Coleoptera sp. 1 |  | 10 |
|  | *Corophium* sp. |  | 11 |
|  | *Crangon septemspinosa* |  | 12 |
|  | *Cyathura* sp. |  | 13 |
|  | *Cyathura polita* |  | 14 |
|  | Dulichopidae sp. 1 |  | 15 |
|  | *Gammarus* sp. 1 |  | 16 |
|  | Harpacticoid sp. 1 |  | 17 |
|  | *Hobsonia florida* |  | 18 |
|  | *Hydrobia minuta* |  | 19 |
|  | *Melampus bidentatus* |  | 20 |
|  | Nematoda sp. 1 |  | 21 |
|  | Oligochaeta sp. 1 |  | 22 |
|  | *Orchestia* sp. |  | 23 |
|  | Ostracoda sp. 1 |  | 24 |
|  | *Palaeomonetes pugio* |  | 25 |
|  | *Philoscia* sp. |  | 26 |
|  | *Philoscia vittata* |  | 27 |
|  | *Polydora ligni* |  | 28 |
|  | *Rhithropanopeus harrisii* |  | 29 |
|  | Spionidae sp. 1 |  | 30 |
|  | Tabanidae sp. 1 |  | 31 |
|  | *Fishes* |  |  |
|  | *Alosa pseudoharengus* | Alewife | 32 |
|  | *Anguilla rostrata* | American eel | 33 |
|  | *Alosa sapidissima* | American shad | 34 |
|  | *Brevoortia tyrannus* | Atlantic menhaden | 35 |
|  | *Menidia menidia* | Atlantic silverside | 36 |
|  | *Anchoa mitchilli* | Bay anchovy | 37 |
|  | *Pomoxis nigromaculatus* | Black crappie | 38 |
|  | *Alosa aestivalis* | Blueback herring | 39 |
|  | *Pomatomus saltatrix* | Bluefish | 40 |
|  | *Ameiurus nebulosus* | Brown bullhead | 41 |
|  | *Cyprinus carpio carpio* | Carp | 42 |
|  | *Anodontostoma chacunda* | Gizzard shad | 43 |
|  | *Menidia beryllina* | Inland silverside | 44 |
|  | *Fundulus heteroclitus* | Mummichog | 45 |
|  | *Syngnathus fuscus* | Northern pipefish | 46 |
|  | *Lepomis megalotis* | Pumpkinseed | 47 |
|  | *Leiostomus xanthurus* | Spot | 48 |
|  | *Fundulus majalis* | Striped killifish | 49 |
|  | *Morone saxatilis* | Striped bass | 50 |
|  | *Gasterosteus aculeatus aculeatus* | Threespine stickleback | 51 |
|  | *Cynoscion regalis* | Weakfish | 52 |
|  | *Morone americana* | White perch | 53 |
|  | *Pseudopleuronectes americanus* | Winter flounder | 54 |
|  | *Birds* |  |  |
|  | *Anas rubripes* | American black duck | 55 |
|  | *Carduelis tristis* | American goldfinch | 56 |
|  | *Falco sparverius* | American kestrel | 57 |
|  | *Turdus migratorius* | American robin | 58 |
|  | *Anas americana* | American wigeon | 59 |
|  | *Spizella arborea* | American tree sparrow | 60 |
|  | *Hirundo rustica* | Barn Swallow | 61 |
|  | *Pluvialis squatarola* | Black-bellied plover | 62 |
|  | *Ceryle alcyon* | Belted kingfisher | 63 |
|  | *Molothrus ater* | Brown-headed cowbird | 64 |
|  | *Riparia riparia* | Bank swallow | 65 |
|  | *Rynchops niger* | Black skimmer | 66 |
|  | *Branta bernicla* | Brant | 67 |
|  | *Branta canadensis* | Canada goose | 68 |
|  | *Calidris sp.* | *Calidris* sp. | 69 |
|  | *Bombycilla cedrorum* | Cedar waxwing | 70 |
|  | *Chaetura pelagica* | Chimney swift | 71 |
|  | *Quiscalus quiscula* | Common grackle | 72 |
|  | *Mergus merganser* | Common merganser | 73 |
|  | *Corvus sp.* | *Corvus* sp. | 74 |
|  | *Geothlypis trichas* | Common yellowthroat | 75 |
|  | *Phalacrocorax auritus* | Double-crested cormorant | 76 |
|  | *Calidris alpina* | Dunlin | 77 |
|  | *Sturnus vulgaris* | European starling | 78 |
|  | *Sterna forsteri* | Forster’s tern | 79 |
|  | *Anas strepera* | Gadwall | 80 |
|  | *Larus marinus* | Great black-backed gull | 81 |
|  | *Ardea herodias* | Great blue heron | 82 |
|  | *Dumetella carolinensis* | Gray catbird | 83 |
|  | *Ardea alba* | Great egret | 84 |
|  | *Butorides virescens* | Green heron | 85 |
|  | *Anas crecca* | Green-winged teal | 86 |
|  | *Larus argentatus* | Herring gull | 87 |
|  | *Lophodytes cucullatus* | Hooded merganser | 88 |
|  | *Passer domesticus* | House sparrow | 89 |
|  | *Charadrius vociferus* | Killdeer | 90 |
|  | *Larus atricilla* | Laughing gull | 91 |
|  | *Anas platyrhynchos* | Mallard | 92 |
|  | *Cistothorus palustris* | Marsh wren | 93 |
|  | *Zenaida macroura* | Mourning dove | 94 |
|  | *Cardinalis cardinalis* | Northern cardinal | 95 |
|  | *Circus cyaneus* | Northern harrier | 96 |
|  | *Mimus polyglottos* | Northern mockingbird | 97 |
|  | *Anas clypeata* | Northern shoveler | 98 |
|  | *Stelgidopteryx serripennis* | Northern rough-winged swallow | 99 |
|  | *Pandion haliaetus* | Osprey | 100 |
|  | *Dendroica palmarum* | Palm warbler | 101 |
|  | *Falco peregrinus* | Peregrine falcon | 102 |
|  | *Larus delawarensis* | Ring-billed gull | 103 |
|  | *Regulus calendula* | Ruby-crowned kinglet | 104 |
|  | *Phasianus colchicus* | Ring-necked pheasant | 105 |
|  | *Buteo jamaicensis* | Red-tailed hawk | 106 |
|  | *Agelaius phoeniceus* | Red-winged blackbird | 107 |
|  | *Passerculus sandwichensis* | Savannah sparrow | 108 |
|  | *Charadrius semipalmatus* | Semipalmated plover | 109 |
|  | *Egretta thula* | Snowy egret | 110 |
|  | *Melospiza melodia* | Song sparrow | 111 |
|  | *Actitis macularius* | Spotted Sandpiper | 112 |
|  | *Melospiza georgiana* | Swamp sparrow | 113 |
|  | *Tringa spp.* | *Tringa* spp. | 114 |
|  | *Tachycineta bicolor* | Tree swallow | 115 |
|  | *Empidonax traillii* | Willow Flycatcher | 116 |
|  | *Zonotrichia albicollis* | White-throated sparrow | 117 |
|  | *Dendroica petechia* | Yellow warbler | 118 |
|  | *Dendroica coronata* | Yellow-rumped warbler | 119 |
|  | *Basal food items* |  |  |
|  | Terrestrial and aquatic detritus |  | D |
|  | Micro- and macro-algae |  | A |
|  | Producers |  | P |
| Harrier Meadow Marsh | *Benthic invertebrates* |  |  |
|  | *Callinectes* *sapidus* |  | 1 |
|  | *Idotea* sp. |  | 2 |
|  | *Rhithropanopeus harrissii* |  | 3 |
|  | Oligochaeta sp. 1 |  | 4 |
|  | Nematoda sp. 1 |  | 5 |
|  | *Manayunkia* *aestuarina* |  | 6 |
|  | Chironomidae sp. 1 |  | 7 |
|  | Copepoda sp. 1 |  | 8 |
|  | Anthozoa sp. 1 |  | 9 |
|  | *Capitella* sp. |  | 10 |
|  | Ostracoda sp. 1 |  | 11 |
|  | *Congeria* sp. |  | 12 |
|  | *Corophium* sp. |  | 13 |
|  | *Crangon* sp. |  | 14 |
|  | *Palaeomonetes* sp. |  | 15 |
|  | *Fishes* |  |  |
|  | *Alosa pseudoharengus* | Alewife | 16 |
|  | *Anguilla rostrata* | American eel | 17 |
|  | *Brevoortia tyrannus* | Atlantic menhaden | 18 |
|  | *Menidia menidia* | Atlantic silverside | 19 |
|  | *Alosa aestivalis* | Blueback herring | 20 |
|  | *Pomatomus saltatrix* | Bluefish | 21 |
|  | *Cyprinus carpio carpio* | Carp | 22 |
|  | *Caranx hippos* | Crevalle jack | 23 |
|  | *Anodontostoma chacunda* | Gizzard shad | 24 |
|  | *Selene vomer* | Lookdown | 25 |
|  | *Fundulus heteroclitus* | Mummichog | 26 |
|  | *Urophycis regia* | Spotted hake | 27 |
|  | *Morone saxatilis* | Striped bass | 28 |
|  | *Fundulus majalis* | Striped killifish | 29 |
|  | *Cynoscion regalis* | Weakfish | 30 |
|  | *Morone americana* | White perch | 31 |
|  | *Pseudopleuronectes americanus* | Winter flounder | 32 |
|  | *Birds* |  |  |
|  | *Carduelis tristis* | American goldfinch | 33 |
|  | *Anas rubripes* | American black duck | 34 |
|  | *Fulica americana* | American coot | 35 |
|  | *Falco sparverius* | American kestrel | 36 |
|  | *Turdus migratorius* | American robin | 37 |
|  | *Hirundo rustica* | Barn swallow | 38 |
|  | *Pittasoma michleri* | Black-crowned night heron | 39 |
|  | *Icterus galbula* | Baltimore oriole | 40 |
|  | *Poecile atricapillus* | Blackcapped chickadee | 41 |
|  | *Ceryle alcyon* | Belted kingfisher | 42 |
|  | *Molothrus ater* | Brownheaded cowbird | 43 |
|  | *Cyanocitta cristata* | Bluejay | 44 |
|  | *Rynchops niger* | Black skimmer | 45 |
|  | *Toxostoma rufum* | Brown thrasher | 46 |
|  | *Anas discors* | Blue winged teal | 47 |
|  | *Branta canadensis* | Canada goose | 48 |
|  | *Calidris spp.* | Calidris species | 49 |
|  | *Quiscalus quiscula* | Common grackle | 50 |
|  | *Mergus merganser* | Common merganser | 51 |
|  | *Corvus spp.* | Corvus spp. | 52 |
|  | *Geothlypis trichas* | Common yellowthroat | 53 |
|  | *Phalacrocorax auritus* | Double-crested cormorant | 54 |
|  | *Junco hyemalis* | Dark-eyed junco | 55 |
|  | *Sturnus vulgaris* | European starling | 56 |
|  | *Sayornis phoebe* | Eastern phoebe | 57 |
|  | *Sterna forsteri* | Forster's tern | 58 |
|  | *Anas strepera* | Gadwall | 59 |
|  | *Larus marinus* | Great black-backed gull | 60 |
|  | *Ardea herodias* | Great blue heron | 61 |
|  | *Dumetella carolinensis* | Gray catbird | 62 |
|  | *Butorides virescens* | Green heron | 63 |
|  | *Anas crecca* | Green-winged teal | 64 |
|  | *Ardea alba* | Great egret | 65 |
|  | *Lophodytes cucullatus* | Hooded merganser | 66 |
|  | *Passer domesticus* | House sparrow | 67 |
|  | *Larus argentatus* | Herring gull | 68 |
|  | *Charadrius vociferus* | Killdeer | 69 |
|  | *Larus atricilla* | Laughing gull | 70 |
|  | *Limnodromus spp.* | Limnodromus spp. | 71 |
|  | *Anas platyrhynchos* | Mallard | 72 |
|  | *Cistothorus palustris* | Marsh wren | 73 |
|  | *Zenaida macroura* | Mourning dove | 74 |
|  | *Cygnus olor* | Mute swan | 75 |
|  | *Cardinalis cardinalis* | Northern cardinal | 76 |
|  | *Colaptes auratus* | Northern flicker | 77 |
|  | *Circus cyaneus* | Northern harrier | 78 |
|  | *Anas acuta* | Northern pintail | 79 |
|  | *Anas clypeata* | Northern shoveler | 80 |
|  | *Stelgidopteryx serripennis* | Northern rough-winged swallow | 81 |
|  | *Mimus polyglottos* | Northern mockingbird | 82 |
|  | *Pandion haliaetus* | Osprey | 83 |
|  | *Falco peregrinus* | Peregrine falcon | 84 |
|  | *Larus delawarensis* | Ring-billed gull | 85 |
|  | *Columba livia* | Rock pigeon | 86 |
|  | *Aythya collaris* | Ring-necked duck | 87 |
|  | *Phasianus colchicus* | Ring-necked pheasant | 88 |
|  | *Buteo jamaicensis* | Red-tailed hawk | 89 |
|  | *Oxyura jamaicensis* | Ruddy duck | 90 |
|  | *Agelaius phoeniceus* | Red-winged blackbird | 91 |
|  | *Charadrius semipalmatus* | Semipalmated plover | 92 |
|  | *Passerculus sandwichensis* | Savannah sparrow | 93 |
|  | *Chen caerulescens* | Snow goose | 94 |
|  | *Porzana carolina* | Sora | 95 |
|  | *Egretta thula* | Snowy egret | 96 |
|  | *Melospiza melodia* | Song sparrow | 97 |
|  | *Actitis macularius* | Spotted sandpiper | 98 |
|  | *Melospiza georgiana* | Swamp sparrow | 99 |
|  | *Tringa spp.* | Tringa species (shanks) | 100 |
|  | *Egretta tricolor* | Tri-colored heron | 101 |
|  | *Tachycineta bicolor* | Tree swallow | 102 |
|  | *Rallus limicola* | Virginia rail | 103 |
|  | *Zonotrichia leucophrys* | White-crowned sparrow | 104 |
|  | *Empidonax traillii* | Willow fly-catcher | 105 |
|  | *Wilsonia pusilla* | Wilson's phalarope | 106 |
|  | *Aix sponsa* | Wood duck | 107 |
|  | *Dendroica petechia* | Yellow warbler | 108 |
|  | *Dendroica coronata* | Yellow-rumped warbler | 109 |
|  | *Basal food items* |  |  |
|  | Terrestrial and aquatic detritus |  | D |
|  | Micro- and macro-algae |  | A |
|  | Producers |  | P |
| Oritani Marsh | *Benthic Invertebrates* |  |  |
|  | *Hobsonia florida* |  | 1 |
|  | *Streblospio benedicti* |  | 2 |
|  | *Scolecolepides virides* |  | 3 |
|  | *Nereis succinea* |  | 4 |
|  | Oligochaeta sp. 1 |  | 5 |
|  | *Palmacorixa* sp. |  | 6 |
|  | Chironomidae sp. 1 |  | 7 |
|  | *Balanus improvisus* |  | 8 |
|  | *Cyathura polita* |  | 9 |
|  | *Idotea* sp. |  | 10 |
|  | *Rhithropanopeus harisii* |  | 11 |
|  | *Uca minax* |  | 12 |
|  | *Gammarus* sp. |  | 13 |
|  | *Littorina* sp. |  | 14 |
|  | *Melampus bidentatus* |  | 15 |
|  | *Macoma balthica* |  | 16 |
|  | Sipuncoloidea sp. 1 |  | 17 |
|  | Nematoda sp. 1 |  | 18 |
|  | Nemertea sp. 1 |  | 19 |
|  | *Callinectes sapidus* |  | 20 |
|  | *Crangon septemspinosa* |  | 21 |
|  | *Palaemonetes* sp. |  | 22 |
|  | *Fishes* |  |  |
|  | *Alosa sapidissima* | American shad | 23 |
|  | *Alosa pseudoharengus* | Alewife | 24 |
|  | *Anguilla rostrata* | American eel | 25 |
|  | *Brevoortia tyrannus* | Atlantic menhaden | 26 |
|  | *Menidia menidia* | Atlantic silverside | 27 |
|  | *Anchoa mitchilli* | Bay anchovy | 28 |
|  | *Alosa aestivalis* | Blueback herring | 29 |
|  | *Pomatomus saltatrix* | Bluefish | 30 |
|  | *Ameiurus nebulosus* | Brown bullhead | 31 |
|  | *Cyprinus carpio carpio* | Carp | 32 |
|  | *Caranx hippos* | Crevalle jack | 33 |
|  | *Anodontostoma chacunda* | Gizzard shad | 34 |
|  | *Trinectes maculatus* | Hogchoker | 35 |
|  | *Menidia beryllina* | Inland silverside | 36 |
|  | *Fundulus heteroclitus* | Mummichog | 37 |
|  | *Urophycis regia* | Spotted hake | 38 |
|  | *Leiostomus xanthurus* | Spot | 39 |
|  | *Morone saxatilis* | Striped bass | 40 |
|  | *Fundulus majalis* | Striped killifish | 41 |
|  | *Gasterosteus aculeatus aculeatus* | Threespined stickleback | 42 |
|  | *Cynoscion regalis* | Weakfish | 43 |
|  | *Pseudopleuronectes americanus* | Winter flounder | 44 |
|  | *Morone americana* | White perch | 45 |
|  | *Birds* |  |  |
|  | *Tachycineta bicolor* | Tree swallow | 46 |
|  | *Spizella passerina* | Chipping sparrow | 47 |
|  | *Phasianus colchicus* | Ring-necked pheasant | 48 |
|  | *Turdus migratorius* | American robin | 49 |
|  | *Ardea alba* | Great egret | 50 |
|  | *Anas platyrhynchos* | Mallard | 51 |
|  | *Circus cyaneus* | Northern harrier | 52 |
|  | *Melospiza melodia* | Song sparrow | 53 |
|  | *Melospiza georgiana* | Swamp sparrow | 54 |
|  | *Geothlypis trichas* | Common yellowthroat | 55 |
|  | *Larus argentatus* | Herring gull | 56 |
|  | *Cistothorus palustris* | Marsh wren | 57 |
|  | *Agelaius phoeniceus* | Red-winged blackbird | 58 |
|  | *Falco sparverius* | American kestrel | 59 |
|  | *Quiscalus quiscula* | Common grackle | 60 |
|  | *Cardinalis cardinalis* | Northern cardinal | 61 |
|  | *Corvus spp.* | American crow | 62 |
|  | *Anas discors* | Blue-winged teal | 63 |
|  | *Phalacrocorax auritus* | Double-crested cormorant | 64 |
|  | *Sturnus vulgaris* | European starling | 65 |
|  | *Cyanocitta cristata* | Blue jay | 66 |
|  | *Passerculus sandwichensis* | Savannah sparrow | 67 |
|  | *Basal food items* |  |  |
|  | Terrestrial and aquatic detritus |  | D |
|  | Micro- and macro-algae |  | A |
|  | Producers |  | P |
| Secaucus High School Marsh | *Benthic Invertebrates* |  |  |
|  | Oligochaeta sp. 1 |  | 1 |
|  | Nematoda sp. 1 |  | 2 |
|  | *Chironomus* sp. |  | 3 |
|  | *Glycera* sp. |  | 4 |
|  | Empididae sp. 1 |  | 5 |
|  | Ceratopogonidae sp. 1 |  | 6 |
|  | Tipulidae sp. 1 |  | 7 |
|  | *Corophium* sp. |  | 8 |
|  | *Callinectes sapidus* |  | 9 |
|  | *Rhithropanopeous harisii* |  | 10 |
|  | *Crangon septemspinosa* |  | 11 |
|  | *Palaemonetes* sp. |  | 12 |
|  | *Fishes* |  |  |
|  | *Alosa pseudoharengus* | Alewife | 13 |
|  | *Anguilla rostrata* | American eel | 14 |
|  | *Brevoortia tyrannus* | Atlantic menhaden | 15 |
|  | *Menidia menidia* | Atlantic silverside | 16 |
|  | *Alosa aestivalis* | Blueback herring | 17 |
|  | *Ameiurus nebulosus* | Brown bullhead | 18 |
|  | *Cyprinus carpio carpio* | Carp | 19 |
|  | *Anodontostoma chacunda* | Gizzard shad | 20 |
|  | *Menidia beryllina* | Inland silverside | 21 |
|  | *Fundulus heteroclitus* | Mummichog | 22 |
|  | *Lepomis gibbosus* | Pumpkinseed | 23 |
|  | *Morone saxatilis* | Striped bass | 24 |
|  | *Fundulus majalis* | Striped killifish | 25 |
|  | *Gasterosteus aculeatus aculeatus* | Threespine stickleback | 26 |
|  | *Cynoscion regalis* | Weakfish | 27 |
|  | *Morone americana* | White perch | 28 |
|  | *Pseudopleuronectes americanus* | Winter flounder | 29 |
|  | *Birds* |  |  |
|  | *Anas rubripes* | American black duck | 30 |
|  | *Branta canadensis* | Canada goose | 31 |
|  | *Anas strepera* | Gadwall | 32 |
|  | *Anas platyrhynchos* | Mallard | 33 |
|  | *Larus marinus* | Greater black-backed gull | 34 |
|  | *Tringa melanoleuca* | Greater yellowlegs | 35 |
|  | *Larus argentatus* | Herring gull | 36 |
|  | *Calidris minutilla* | Least sandpiper | 37 |
|  | *Larus delawarensis* | Ring-billed gull | 38 |
|  | *Calidris alba* | Sanderling | 39 |
|  | *Pittasoma michleri* | Black-crowned night heron | 40 |
|  | *Ardea herodias* | Great blue heron | 41 |
|  | *Ardea alba* | Great egret | 42 |
|  | *Butorides virescens* | Green heron | 43 |
|  | *Botaurus lentiginosus* | American bittern | 44 |
|  | *Ixobrychus exilis* | Least bittern | 45 |
|  | *Egretta thula* | Snowy egret | 46 |
|  | *Zenaida macroura* | Mourning dove | 47 |
|  | *Ceryle alcyon* | Belted kingfisher | 48 |
|  | *Falco sparverius* | American kestrel | 49 |
|  | *Circus cyaneus* | Northern harrier | 50 |
|  | *Pandion haliaetus* | Osprey | 51 |
|  | *Rallus limicola* | Virginia rail | 52 |
|  | *Corvus spp.* | American crow | 53 |
|  | *Carduelis tristis* | American goldfinch | 54 |
|  | *Turdus migratorius* | American robin | 55 |
|  | *Spizella arborea* | American tree sparrow | 56 |
|  | *Hirundo rustica* | Barn swallow | 57 |
|  | *Dendroica striata* | Blackpoll warbler | 58 |
|  | *Cyanocitta cristata* | Blue jay | 59 |
|  | *Spizella passerina* | Chipping sparrow | 60 |
|  | *Quiscalus quiscula* | Common grackle | 61 |
|  | *Geothlypis trichas* | Common yellowthroat | 62 |
|  | *Sturnus vulgaris* | European starling | 63 |
|  | *Spizella pusilla* | Field sparrow | 64 |
|  | *Dumetella carolinensis* | Grey catbird | 65 |
|  | *Passer domesticus* | House sparrow | 66 |
|  | *Junco hyemalis* | Junco | 67 |
|  | *Cistothorus palustris* | Marsh wren | 68 |
|  | *Mimus polyglottos* | Northern mockingbird | 69 |
|  | *Icterus galbula* | Northern oriole | 70 |
|  | *Dendroica palmarum* | Palm warbler | 71 |
|  | *Dendroica pinus* | Pine warbler | 72 |
|  | *Agelaius phoeniceus* | Red-winged blackbird | 73 |
|  | *Pipilo erythrophthalmus* | Rufous-sided towhee | 74 |
|  | *Passerculus sandwichensis* | Savannah sparrow | 75 |
|  | *Ammodramus caudacutus* | Sharp-tailed sparrow | 76 |
|  | *Melospiza melodia* | Song sparrow | 77 |
|  | *Melospiza georgiana* | Swamp sparrow | 78 |
|  | *Tachycineta bicolor* | Tree swallow | 79 |
|  | *Zonotrichia leucophrys* | White-throated sparrow | 80 |
|  | *Dendroica petechia* | Yellow warbler | 81 |
|  | *Dendroica coronata* | Yellow-rumped warbler | 82 |
|  | *Phalacrocorax auritus* | Double-crested cormorant | 83 |
|  | *Colaptes auratus* | Northern flicker | 84 |
|  | *Basal food items* |  |  |
|  | Terrestrial and aquatic detritus |  | D |
|  | Micro- and macro-algae |  | A |
|  | Producers |  | P |
