# Supplementary material for: Host Centrality in Food Web Networks Determines Parasite Diversity
Source: PLoS One. 2011 Oct 25;6(10):e26798. doi: 10.1371/journal.pone.0026798 (PMC3201966; doi:10.1371/journal.pone.0026798)
Supplement: Table S2 — Life cycle characteristics of select parasites in the Meadowlands estuary complex. Parasite species marked with a star (*) represent those identified in field collections of Fundulus heteroclitus. (DOC) [file pone.0026798.s004.doc]

Table S2: Life cycle characteristics of select parasites in the Meadowlands estuary complex. Parasite species marked with a star (*) represent those identified in field collections of *Fundulus heteroclitus.*

| **Parasite species** | **Type** | **Intermediate Host 1** | **Intermediate Host 2** | **Definitive Host** |
| --- | --- | --- | --- | --- |
| *Contracaecum* sp.* | Nematode | Copepods Amphipods (2 spp.) | Fish (5 spp.) | Piscivorous birds (7 spp.) |
| *Eustrongylides* sp. | Nematode | Oligochaete (1 spp.) | Fish (1 spp.) | Piscivorous birds (6 spp.) |
| *Paratenuisentis ambiguous** | Acanthocephalan | Amphipods (1 spp.) |  | *Anguilla* *rostrata* |
| *Leptorhynchoides thecatus* | Acanthocephalan | Amphipods (1 spp.) |  | Fish (3 spp.) |
| *Ascocotyle diminuta** | Trematode | Gastropoda (2 spp.) | Fish (2 spp.) | Piscivorous birds (6 spp.) |
| *Posthodiplostomum minimum** | Trematode | Gastropoda (2 spp.) | Fish (3 spp.) | Piscivorous birds (4 spp.) |
| *Schistocephalus solidus* | Cestode | Copepod (1 spp) | Fish (1 spp) | Piscivorous birds (8 spp.) |
| *Proteocephalus* sp. | Cestode | Copepod (1 spp.) |  | Eels, fish (6 spp.) |
| Spirocamellanus sp. | Nematode | Copepod (1 spp.) |  | Fish (4 spp.) |
| Euhaplorchis sp. | Trematode | Gastropoda (1 sp.) | Fish (2 spp.) | Piscivorous birds (13 spp.) |
| Himasthla sp. 1 | Trematode | Gastropoda (1 sp.) | Misc. invertebrates (4 spp.) | Laridae and Charadriidae (7 spp.) |
| Probolocoryphe sp. | Trematode | Gastropoda (1 sp.) | Misc. invertebrates (4 spp.) | Scolopacidae, Laridae, Charadriidae (9 spp.) |
| Himasthla sp. 2 | Trematode | Gastropoda (1 sp.) | Spionid polychaetes (3 spp.) | Anatidae, Scolopacidae, Charadriidae (10 spp.) |
| Renicola sp. | Trematode | Gastropoda (1 sp.) | Fish (2 spp.) | Scolopacidae, Charadriidae, Pandionidae, Laridae, Ardeidae, Cerylidae (14 spp.) |
| Acanthoparyphium sp. | Trematode | Gastropoda (1 sp.) | Misc. invertebrates (4 spp.) | Rallidae, Scolopacidae, Charadriidae (9 spp.) |
| Parorchis sp. | Trematode | Gastropoda (1 sp.) | Misc. invertebrates and fish (7 spp.) | Charadriidae, Phalacrocoracidae, Scolopacidae, Ardeidae, Cerylidae, Laridae (13 spp.) |
| Cloacitrema sp. | Trematode | Gastropoda (1 sp.) | Misc. invertebrates and fish (7 spp.) | Charadriidae, Phalacrocoracidae, Scolopacidae, Ardeidae, Cerylidae, Laridae (12 spp.) |
| Dilepidid sp. | Cestode |  | Fish (2 spp.) | Cerylidae, Ardeidae, Scolopacidae, Phalacrocoracida, Pandionidae (11 spp.) |
| Mesostephanus sp. | Trematode | Gastropoda (1 sp.) | Fish (2 spp.) | Cerylidae, Ardeidae, Scolopacidae, Phalacrocoracida, Pandionidae, Laridae (14 spp.) |
| Pygidiopsoides sp. | Trematode | Gastropoda (1 sp.) | Fish (4 spp.) | Cerylidae, Ardeidae, Scolopacidae, Phalacrocoracida, Pandionidae, Laridae (17 spp.) |
| Microphallid sp. | Trematode | Gastropoda (1 sp.) | Amphipods (2 spp.) | Scolopacidae, Laridae, Charadriidae, Ardeidae (8 spp.) |
| Phocitremoides sp. | Trematode | Gastropoda (1 sp.) | Fish (4 spp.) | Cerylidae, Ardeidae, Scolopacidae, Phalacrocoracida, Pandionidae, Laridae (17 spp.) |
| Renicola sp. 2 | Trematode | Gastropoda (1 sp.) | Fish (2 spp.) | Scolopacidae, Charadriidae, Pandionidae, Laridae, Ardeidae, Cerylidae (14 spp.) |
| Cyathocotylid sp. | Trematode | Gastropoda (1 sp.) | Fish (3 spp.) | Cerylidae, Ardeidae, Scolopacidae, Phalacrocoracida, Pandionidae, Laridae (17 spp.) |
| Stictodora sp. | Trematode | Gastropoda (1 sp.) | Fish (4 spp.) | Cerylidae, Ardeidae, Scolopacidae, Phalacrocoracida, Pandionidae, Laridae (17 spp.) |
