# Supplementary material for: Host Centrality in Food Web Networks Determines Parasite Diversity
Source: PLoS One. 2011 Oct 25;6(10):e26798. doi: 10.1371/journal.pone.0026798 (PMC3201966; doi:10.1371/journal.pone.0026798)
Supplement: Table S3 — Arrangement of Mill Creek, Harrier Meadow, Oritani and Secaucus High School Marsh food webs into group structure by the algorithm proposed by Allesina & Pascual [60]. (DOC) [file pone.0026798.s005.doc]

Table S3: Arrangement of Mill Creek, Harrier Meadow, Oritani and Secaucus High School Marsh food webs into groups of size 1 through 20 by the algorithm proposed by Allesina and Pascual (2009). Those *AIC* values marked with a * represent minimum *AIC*.

| **Groups (*k*):** | *AIC scores Oritani Marsh (unrestored)* | *AIC scores Secaucus Marsh (0 year)* | *AIC scores Harrier Marsh (10 year)* | *AIC scores Mill Creek Marsh (20 year)* |
| --- | --- | --- | --- | --- |
| 1 | 3938.216 | 4500.115 | 8166.891 | 11405.133 |
| 2 | 2984.322 | 3338.351 | 5718.195 | 8081.771 |
| 3 | 2596.172 | 2843.945 | 4694.515 | 7011.132 |
| 4 | 2316.339 | 2512.891 | 4224.433 | 6198.703 |
| 5 | 2136.477 | 2241.238 | 3618.488 | 5472.594 |
| 6 | 1989.983 | 2011.916 | 3182.488 | 4997.905 |
| **7** | 1842.378 | 1830.695 | 2974.971 | 4546.075 |
| 8 | 1732.381 | 1688.711 | 2710.174 | 4254.384 |
| 9 | 1633.975 | 1609.049 | 2442.058 | 4018.755 |
| 10 | 1543.812 | 1539.818 | 2264.517 | 3759.184 |
| 11 | 1477.240 | 1501.421 | 2155.802 | 3543.062 |
| 12 | 1432.574 | 1470.762 | 2027.466 | 3326.204 |
| 13 | 1385.056 | 1440.171 | 1948.568 | 3242.951 |
| 14 | 1380.067 | 1411.697 | 1904.399 | 3174.256 |
| 15 | 1361.204* | 1403.699* | 1895.185 | 3118.203 |
| 16 | 1372.144 | 1410.972 | 1870.390 | 3093.556 |
| 17 | 1388.636 | 1416.781 | 1855.325 | 3071.354 |
| 18 | 1416.735 | 1443.747 | 1851.463* | 3044.921 |
| 19 | 1454.468 | 1480.152 | 1865.241 | 3016.614* |
| 20 | 1500.863 | 1521.987 | 1864.637 | 3021.110 |
