# Supplementary material for: Host Centrality in Food Web Networks Determines Parasite Diversity
Source: PLoS One. 2011 Oct 25;6(10):e26798. doi: 10.1371/journal.pone.0026798 (PMC3201966; doi:10.1371/journal.pone.0026798)
Supplement: Table S4 — Regression tree variable importance determined by the summed reduction in the loss function (e.g. mean squared error) attributed to each variable at each split. Random forest variable importance is determined by calculating the mean square error during each random permutation (n = 1000), and determining the difference between the average value and the prediction error on the out-of-bag data. (DOC) [file pone.0026798.s006.doc]

Table S4: Regression tree variable importance determined by the summed reduction in the loss function (e.g. mean squared error) attributed to each variable at each split. Random forest variable importance is determined by calculating the mean square error during each random permutation (n = 1000), and determining the difference between the average value and the prediction error on the out-of-bag data.

| Model type | Predictor variable | Overall reduction in MSE |
| --- | --- | --- |
| Regression tree | Betweenness | 0.5060049 |
|  | Closeness | 1.6047845 |
|  | Coreness | 1.3072689 |
|  | Degree | 0.8019895 |
|  | Eigenvector | 1.0912676 |
|  | Group | 0.6235651 |
|  | Marsh_diversity | 0.5490112 |
|  | num_predators | 0.4447786 |
|  | num_prey | 0.8194352 |
| Random forest | Betweenness | 23.991370 |
|  | Closeness | 30.525407 |
|  | Coreness | 19.325506 |
|  | Degree | 24.791385 |
|  | Eigenvector | 31.778560 |
|  | Group | 16.366579 |
|  | Marsh_diversity | 8.355787 |
|  | num_predators | 30.889810 |
|  | num_prey | 37.839771 |
